# Surface Contamination by Multidrug‐Resistant Gram‐Negative Bacteria in a Healthcare Facility: Resistance Determinants and Biofilm‐Associated Adhesion

**DOI:** 10.1155/ijm/5734443

**Published:** 2026-05-05

**Authors:** Asmaa Dihmane, Rafik Aniba, Habiba Raqraq, Amina Ressmi, Kaotar Nayme, Mohammed Timinouni, Abouddihaj Barguigua

**Affiliations:** ^1^ Team of Biotechnology and Sustainable Development of Natural Resources, Department of Biology-Geology, Polydisciplinary Faculty, Sultan Moulay Slimane University, Beni Mellal, Morocco, universitesms.com; ^2^ Laboratoire de bactériologie moléculaire, Institut Pasteur du Maroc, Casablanca, Morocco, pasteur.ma; ^3^ Ecole des Hautes Études de Biotechnologie et de Santé (EHEB), Casablanca, Morocco

**Keywords:** antimicrobial resistance genes, biofilm-associated adhesion, carbapenemase and ESBL producers, disinfectant tolerance, environmental persistence, hospital surfaces, multidrug-resistant Gram-negative bacteria

## Abstract

Hospital surfaces represent a major reservoir of multidrug‐resistant Gram‐negative bacteria (MDR‐GNB), which contributes to healthcare‐associated infections. This study characterized the occurrence, resistance determinants, and biofilm‐forming behavior of MDR‐GNB isolated from hospital surfaces in a Moroccan regional hospital. Samples were collected from multiple departments and subjected to phenotypic and molecular analysis to characterize antimicrobial resistance, resistance genes, and adhesion properties. Among the 154 sampled surfaces, 62% were contaminated with Gram‐negative bacilli, predominantly *Acinetobacter baumannii* (39%), *Escherichia coli* (21%), and *Enterobacter cloacae* (11%). Based on molecular analyses, the key resistance genes were *bla*_NDM-1_, *bla*_OXA-48_, *bla*_VIM-1_, and *qacΔ*E1, and 73% of the isolates were multidrug resistant (a multiple antibiotic resistance index ≥ 0.6). The majority of the isolates (72.7%) were weak biofilm producers. The isolates adhered more strongly to hydrophobic materials (polyvinyl chloride and latex) than to hydrophilic glass (*p* < 0.001). Principal component analysis and hierarchical clustering linked antimicrobial resistance, biocide tolerance, and surface colonization. The co‐occurrence of antibiotic‐ and disinfectant‐resistance genes in MDR‐GNB underlies their ability to persist in clinical environments. These findings support risk‐based surface hygiene strategies that incorporate molecular surveillance, the selection of proper materials, and targeted disinfection protocols.

## 1. Introduction

Hospital‐acquired infections (HAIs) are a major global public health concern: They are responsible for millions of preventable cases each year, particularly in low‐ and middle‐income countries [1, 2]. Multidrug‐resistant Gram‐negative bacilli (MDR‐GNB) such as *Acinetobacter baumannii*, *Klebsiella pneumoniae*, and *Pseudomonas aeruginosa* have emerged as one of the most critical groups of hospital‐associated pathogens [3]. These bacteria have an impermeable outer membrane, active efflux systems, and the ability to survive under adverse environmental conditions [4, 5], all of which complicate infection prevention and treatment strategies. Indeed, the World Health Organization (WHO) has listed these species among the “critical priority pathogens” due to their broad resistance to *β*‐lactams, carbapenems, and fluoroquinolones, which considerably limits therapeutic options [6].

Hospitals constitute complex ecological systems where MDR organisms persist and disseminate in high‐touch environments, contributing silently yet significantly to the transmission of HAIs [7]. Environmental surfaces, especially those in direct contact with patients or medical devices, act as underestimated reservoirs of bacterial contaminants [8, 9]. Even when these surfaces are routinely cleaned, they can remain colonized by clinically relevant pathogens, notably Gram‐negative bacilli (GNB), that can survive prolonged periods of desiccation and resist common disinfectants [10, 11]. GNB persist in hospital environments due to a complex interplay between adaptive mechanisms and environmental factors. They express adhesins, outer membrane proteins, and exopolysaccharides that enable the establishment of biofilms, which are multicellular structures that shield microbes from chemical agents and desiccation and foster resistance [12, 13]. The production of extended‐spectrum *β*‐lactamases (ESBLs), carbapenemases, and plasmid‐mediated quinolone resistance (PMQR) genes exacerbates this phenomenon by reducing the efficacy of key antibiotic families [14, 15]. Moreover, disinfectant‐resistance genes such as *qacEΔ1*, *cepA*, and *acrA* encode proteins that participate in efflux mechanisms, resulting in selective pressure that maintains coresistance to antibiotics and biocides [16, 17]. The mechanisms that underlie the concurrent resistance to antibiotics and biocides include efflux regulation, membrane modification, and stress‐response activation [18].

The physicochemical properties of hospital surfaces also play a key role in bacterial adhesion and colonization. Hydrophobic materials, including latex and polyvinyl chloride (PVC), are more favorable for microbial attachment and biofilm development than hydrophilic surfaces such as glass [19, 20]. The type of surface, the environmental conditions, and microbial physiology can interact to enhance persistence, particularly in areas where high‐risk patients are treated, such as intensive care units (ICUs) and surgical wards [21, 22].

In Morocco, although multidrug‐resistant pathogens have been widely reported in clinical isolates [23–25], little is known about their persistence and ecological behavior on hospital surfaces. Given that GNB such as *A. baumannii* and *K. pneumoniae* are currently listed by the WHO among the most critical antimicrobial‐resistant pathogens, and because their outer membrane structure and efflux mechanisms confer superior environmental persistence compared with Gram‐positive species, our study specifically focused on MDR‐GNB as a relevant model for surface contamination and hospital hygiene risk assessment. This lack of data limits the implementation of evidence‐based disinfection and surveillance strategies suited to local contexts. Investigations addressing the environmental dimension of MDR‐GNB circulation are therefore crucial to strengthen infection control programs.

The present study is aimed at filling this gap by systematically investigating the environmental dissemination and persistence of MDR‐GNB on hospital surfaces. Specifically, it seeks to: (i) determine the prevalence and distribution of MDR‐GNB across different wards; (ii) characterize their antimicrobial and disinfectant resistance profiles at both phenotypic and molecular levels; (iii) evaluate their capacity for biofilm formation and surface adhesion; and (iv) explore the correlations between resistance determinants, disinfectant tolerance, and adhesion behavior through multivariate statistical analysis. By integrating these complementary approaches, this study provides a comprehensive view of the ecological and molecular mechanisms underpinning the persistence of MDR‐GNB in the hospital environment and delivers actionable insights for improving disinfection strategies and antimicrobial stewardship in Moroccan healthcare settings.

## 2. Materials and Methods

### 2.1. Study Site

From April 2022 to January 2023, a prospective descriptive study was conducted at Beni Mellal Regional Hospital, a 420‐bed healthcare facility located in the Béni Mellal–Khénifra region of Morocco. The hospital includes a wide range of clinical services and operates as a referral center for both urban and rural populations.

Sampling was performed in 12 distinct services, selected based on criteria such as high patient density, frequency of invasive procedures, intensity of medical care, and increased potential for environmental contamination. These services included internal medicine, endocrinology and gastroenterology, pediatrics, hemodialysis, pediatric surgery, urology, trauma and neurosurgery, ICU, maternity, oncology, sterilization, and general surgery.

All procedures were carried out in accordance with ethical regulations, and the study received approval from the Institutional Ethics Committee under the Authorization Number: A‐RS‐255/2021. A detailed overview of the hospital services included in the study and the corresponding types of environmental surfaces sampled is provided in Table 1, offering a functional categorization based on patient proximity and risk of contamination.

**Table 1:** Distribution of hospital services and the types of environmental surfaces sampled. Functional categories are based on their proximity to patients, frequency of contact, or role in care delivery.

Hospital department	Representative surfaces sampled	Functional category
Internal medicine	Beds, care carts, and wall switches	Moderate‐risk, patient‐near areas
Gastroenterology	Beds, curtains, and bedside tables	Patient‐contact and surrounding zones
Pediatrics	Beds, care carts, and windows	Pediatric patient‐care areas
Hemodialysis	Beds, dialysis machines, and care carts	High‐use equipment surfaces
Pediatric surgery	Beds, medical equipment, and care carts	High‐touch surgical surfaces
Urology	Beds, care carts, and medical devices	Urologic care areas
Trauma and neurosurgery	Beds, benches, and door handles	High‐contact trauma‐related areas
Intensive care unit	Beds, equipment, and windows	Critical‐care, high‐touch zones
Maternity	Beds, curtains, and bedside tables	Maternal care contact surfaces
Oncology	Beds, benches, and door handles	Immunocompromised care areas
Sterilization	Autoclaves, trolleys, and oxygen sources	Instrument decontamination zones
General surgery	Beds, benches, and walls	Preoperative and postoperative surgical areas

### 2.2. Sampling

After routine biocleaning procedures and in the absence of human activity, surface sampling was performed using the swabbing technique. Sterile swabs were premoistened in a sterile isotonic solution containing a detergent‐neutralizing agent. For each flat surface, the swabs were applied over a defined 25 cm^2^ area in close parallel striations with slight rotation, followed by perpendicular striations, in accordance with ISO/DIS 14698‐1 standards. Inclusion criteria comprised inanimate, high‐touch clinical surfaces located in patient‐care and high‐risk units such as intensive care, surgery, sterilization, and diagnostic areas. Eligible surfaces were those frequently contacted by patients or healthcare workers (e.g., bed rails, door handles, infusion pumps, bedside tables, or medical trolleys) and were sampled immediately after the standard cleaning cycle but before the next patient admission. Exclusion criteria included administrative or decorative areas, nonclinical equipment, visibly damaged or wet materials, and any surface recently exposed to experimental disinfection testing.

To ensure internal consistency, low‐contact reference areas within the same wards (e.g., upper walls or remote equipment stands > 2 m from patient contact) were used as environmental controls to assess baseline contamination under identical sampling conditions. Sampling was repeated on different days and in different hospital wards to improve representativeness.

Collected samples were immediately transferred to the regional hospital′s microbiology laboratory in sterile tubes containing Brain Heart Infusion (BHI) broth (Biokar, Beauvais, France) and incubated at 37°C for 24–48 h.

In this study, “colonization” refers to the stable or reproducible recovery of MDR‐GNB from a given surface, typically associated with biofilm formation and/or persistence‐related genes (*qacΔE1* and *acrA*), whereas “contamination” denotes a transient, nonreproducible recovery without evidence of persistence. These definitions were applied consistently to differentiate enduring environmental colonization from incidental contamination.

### 2.3. Counting Colonies

In the laboratory, every swab was aseptically removed from the test tube and spread onto plate count agar (PCA, Biokar, Beauvais, France). Plates were incubated for 48 h at 37°C. Recovered colony‐forming units (CFU) were counted from every plate, and colonies in every plate were expressed as CFU per 25 cm^2^.

### 2.4. Isolation and Phenotypic Identification of GNB

To isolate GNB, swabs were inoculated onto MacConkey agar and Cetrimide agar (Oxoid, United Kingdom), then incubated at 37°C ± 1°C for 24 h. Distinct colonies with characteristic morphology were subsequently subcultured onto fresh media and incubated under the same conditions for 18–24 h to ensure purity.

Phenotypic identification of purified isolates was based on cultural appearance, Gram staining, and standard biochemical and physiological tests. Final confirmation was performed using the API 20E and API 20NE identification system (bioMérieux, France). Positive and negative control strains were included systematically to validate the accuracy and reliability of the biochemical identification procedures, in accordance with international guidelines for microbial diagnostics.

### 2.5. Antibiotic Susceptibility Test

Antibiotic susceptibility was assessed via disk diffusion on Mueller–Hinton medium (MH, Biokar, Beauvais), in accordance with the European Committee on Antimicrobial Susceptibility Testing guidelines (EUCAST, Version 12.0, 2022). A broad panel of antibiotics was selected, covering multiple antimicrobial classes relevant to the treatment of Gram‐negative infections. The antibiotics tested included:

• *β*‐lactams: ticarcillin (75 *μ*g), ticarcillin–clavulanic acid (85 *μ*g), piperacillin (100 *μ*g), piperacillin–tazobactam (36 *μ*g), ampicillin (10 *μ*g), amoxicillin–clavulanic acid (30 *μ*g), cephalotin (30 *μ*g), ceftriaxone (30 *μ*g), cefotaxime (30 *μ*g), ceftazidime (30 *μ*g), cefoxitin (30 *μ*g), cefepime (30 *μ*g), and aztreonam (30 *μ*g);
• Carbapenems: meropenem (10 *μ*g), ertapenem (10 *μ*g), and imipenem (10 *μ*g);
• Aminoglycosides: gentamicin (10 *μ*g), tobramycin (10 *μ*g), and amikacin (10 *μ*g);
• Fluoroquinolones and quinolones: ciprofloxacin (5 *μ*g), nalidixic acid (30 *μ*g), and levofloxacin (5 *μ*g);
• Tetracyclines: minocycline (30 *μ*g) and tetracycline (30 *μ*g);
• Folate pathway inhibitors: trimethoprim–sulfamethoxazole (25 *μ*g);
• Phenicols: chloramphenicol (30 *μ*g).

To ensure methodological reliability, standardized control strains—*Escherichia coli* ATCC 25922 and *P. aeruginosa* ATCC 27853 (American Type Culture Collection, Manassas, United States)—were systematically included, following EUCAST‐recommended procedures.

Isolates were classified as MDR if they exhibited resistance to at least one antimicrobial agent in three or more distinct classes. The multiple antibiotic resistance (MAR) index was calculated for each isolate using the following formula:

MAR index=Number of antibiotics to which the isolate is resistant Total number of antibiotics tested

### 2.6. ESBL Screening Test

A synergy test was performed on MH agar by placing disks containing ceftazidime, cefepime, cefotaxime, or aztreonam at a 30‐mm distance (center‐to‐center) from a clavulanate disk. ESBL production was inferred when reduced susceptibility to these antibiotics was associated with a distinct enlargement of the inhibition zone adjacent to clavulanate. *K. pneumoniae* ATCC 700603 and *E. coli* ATCC 25922 (American Type Culture Collection, Manassas, United States) were used as positive and negative controls, respectively.

### 2.7. Detection of Carbapenemase Production

Strains of GNB exhibiting resistance against at least one carbapenem antibiotic were subsequently screened for carbapenemase production using phenotypic methods. Carbapenemase activity was assessed via the modified carbapenem inactivation method (mCIM), according to the Clinical and Laboratory Standards Institute (CLSI) guidelines. A suspension of the test isolate was prepared by transferring a 1‐*μ*L loopful of culture from MacConkey agar into 2 mL of Trypticase Soy Broth (TSB) (Oxoid, Hampshire, United Kingdom), into which a 10‐*μ*g meropenem disk was immersed. The mixture was incubated at 35°C for 4 h (±15 min). In parallel, MH Agar was inoculated with a 0.5 McFarland suspension of *E. coli* ATCC 29522. The meropenem disk was subsequently retrieved from the TSB suspension and placed onto MH Agar, followed by incubation at 35°C ± 2°C for 18–24 h. After incubation, the diameter of the inhibition zone surrounding the disk was measured to determine carbapenemase production. A positive result was defined by an inhibition zone of 6–15 mm, or by the presence of pinpoint colonies in a 16–18‐mm zone, whereas *E. coli* ATCC 29522 showed no growth inhibition.

### 2.8. Standard Biofilm Formation Assay

Biofilm formation was quantified using the standard microtiter plate assay with polystyrene wells following previously established protocols [26]. Briefly, 200 *μ*L of overnight cultures grown in BHI broth were inoculated into each well and incubated statically at 37°C for 24 h. Wells were then washed three times with phosphate‐buffered saline (PBS) to remove nonadherent cells. Adherent biofilms were fixed with methanol for 15 min, stained with 0.1% crystal violet solution for 15 min, and gently rinsed with distilled water. The bound dye was solubilized using 200 *μ*L of 95% ethanol. The optical density (OD) was read at 590 nm using a micro‐ELISA reader, and biofilm production was classified relative to the negative control (ODc), as follows:

• Nonbiofilm producers: OD < ODc
• Weak biofilm producers: ODc < OD ≤ 2 × ODc
• Moderate biofilm producers: 2 × ODc < OD ≤ 4 × ODc
• Strong biofilm producers: OD > 4 × ODc

Negative control wells (uninoculated BHI medium) were included in each assay plate to calculate ODc, and all strains were tested in triplicate across three independent experiments. Only results with intra‐assay and interassay coefficients of variation (CV) below 15% were included in the final dataset.

### 2.9. Material‐Dependent Adhesion Analysis

The materials used in this study—stainless steel beads (2 mm diameter), glass beads (2 mm), PVC coupons (5 × 5 mm), latex coupons (5 × 5 mm), and polystyrene coupons (5 × 5 mm)—are representative of those commonly encountered on hospital surfaces. Each material was employed in the form and finish present in our facility. Their physicochemical characteristics, including surface roughness, hydrophobicity, and surface energy, were obtained from manufacturer‐provided technical data sheets and are summarized in Table 2.

**Table 2:** Physicochemical characteristics of materials used for bacterial adhesion assays.

Material	Surface roughness (Ra, *μ*m)	Water contact angle (°)	Surface energy (dynes/cm)	Surface property
Stainless steel	0.2 *μ*m	88°	~90 (estimated)	Weakly hydrophilic
Glass	0.5 *μ*m	25°	~375	Strongly hydrophilic
Polyvinyl chloride	0.3 *μ*m	85.6°	37.9	Weakly hydrophobic
Latex	0.6 *μ*m	95°	~40	Hydrophobic
Polystyrene	0.2 *μ*m	92°	36	Hydrophobic

Prior to use, materials were sterilized and placed in 96‐well microplates, then inoculated with standardized bacterial suspensions following the previously described protocol. After 24‐h incubation at 37°C, each material was gently transferred to sterile microplates for crystal violet staining and subsequent quantification. Material‐specific negative controls were included to normalize OD (OD590 nm) measurements. All experiments were conducted in triplicate under identical conditions (37°C, BHI medium, 24 h incubation). Results were expressed as mean OD590 nm ± standard deviation, normalized to their respective controls.

### 2.10. Determination of the Minimum Inhibitory Concentration (MIC) of Disinfectants

In the present study, we established the antibacterial activity of various disinfectants by the microdilution method in microplates, according to the CLSI guidelines, M07‐11th edition [27]. Serial dilutions of quaternary ammonium derivatives (DDAC 5.1%, NND 2.5%), glutaraldehyde (2%), peracetic acid (4.9%), hydrogen peroxide (23%), sodium hypochlorite (12%), povidone‐iodine (9.6%), and ethanol (96%) were made in 96‐well microplates. A uniform suspension of MDR GNB grown in BHI broth with a modified density of 10^6^ CFU/mL was inoculated in each well. Wells, therefore, were incubated at 37°C for 18–24 h.

MIC is the minimum concentration at which no growth occurs. Three replicate tests in separate plates were performed to determine the MIC for each test. All tests were performed in triplicate on independent plates.

### 2.11. Determination of the Minimum Bactericidal Concentration (MBC)

To determine the MBC, 0.1‐mL aliquots from the MIC wells showing no visible growth were plated onto tryptic soy agar (TSA; Oxoid) and incubated for 48 h at 37°C. The MBC was defined as the lowest disinfectant concentration that resulted in complete bacterial eradication, corresponding to a ≥ 99.99% reduction in viable count from the original inoculum (10^6^ CFU/mL). This threshold aligns with the detection limit of 10 CFU/mL. Each disinfectant–strain pair was tested in triplicate to ensure robustness and reproducibility.

### 2.12. Preparation of DNA Template for PCR

Genomic DNA was extracted using the ISOLATE II Genomic DNA Kit (Bioline, England), following the manufacturer′s protocol. DNA concentrations from ESBL‐ and carbapenemase‐producing isolates were determined using a NanoDrop 2000 spectrophotometer (Thermo Fisher Scientific, United States).

### 2.13. PCR‐Based Screening for ESBL and Carbapenemase Genes

PCR was performed on all isolates identified as ESBL or carbapenemase producers to detect *β*‐lactamase genes. The targeted genes included *bla_SHV_*, *bla_TEM_*, *bla_CTX-M_* (Phylogenetic Groups 1 and 9), *bla_OXA-48_*, *bla_NDM_*, *bla_KPC_*, and *bla_VIM_*, with primer sequences listed in Table 3. Amplification protocols were conducted according to previously validated procedures [23].

**Table 3:** Oligonucleotide primers for molecular detection of antimicrobial and biocides resistance in Gram‐negative bacteria.

Gene	Primer name^a^	Primer sequence (5 ^′^ → 3 ^′^)	Reference
*qnrA*	qnrA(+)	TTCTCACGCCAGGATTTGAG	[23, 26]
	qnrA(−)	TGCCAGGCACAGATCTTGAC	
*qnrB*	qnrB(+)	TGGCGAAAAAAATT(GA)ACAGAA	[23, 26]
	qnrB(−)	GAGCAACGA(TC)GCCTGGTAG	
*qnrS*	qnrS (+)	GACGTGCTAACTTGCGTGAT	[23, 26]
	qnrS(−)	AACACCTCGACTTAAGTCTGA	
*bla*_CTX-M group1_	CTX‐M1(+)	GGTTAAAAAATCACTGCGTC	[23, 26]
	CTX‐M1(−)	TTGGTGACGATTTTAGCCGC	
*bla*_CTX-M group9_	CTX‐M9(+)	ATGGTGACAAAGAGAGTGCA	[23, 26]
	CTX‐M9(−)	CCCTTCGGCGATGATTCTC	
*bla*_TEM_	a‐216(+)	ATAAAATTCTTGAAGACGAAA	[23, 26]
	a‐217(−)	GACAGTTACCAATGCTTAATCA	
*bla*_SHV_	Os‐5(+)	CGCCGGGTTATTCTTATTTGTCGC	[23, 26]
	Os‐6(−)	TCTTTCCGATGCCGCCGCCAGTCA	
*bla*_NDM_	NDM(+)	AATGGAATTGCCAATATTATGC	[24, 26]
	NDM(−)	CGAAAGTCAGGCTGTGTTGC	
*bla*_OXA-48_	OXA‐48(+)	TTGGTGGCATCGATTATCGG	[24, 26]
	OXA‐48(−)	GAGCACTTCTTTTGTGATGGC	
*bla*_VIM_	VIM(+)	TGTGCTKGAGCAAKTCYAGACCG	[24, 26]
	VIM(−)	AGCAAGTTATCTGTATTCTT	
*qacEΔ1*	qacE*Δ*1(+)	AATCCATCCCTGTCGGTGTT	[26, 28]
	qacE*Δ*1(−)	CGCAGCGACTTCCACGATGGGGAT	
*CepA*	CepA(+)	CAACTCCTTCGCCTATCCCG	[26, 29]
	CepA(−)	TCAGGTCAGACCAAACGGCG	
*qacE*	*qacE*(+)	AAGTAATCGCAACATCCG	[26, 30]
	*qacE*(−)	CTACTACACCACTAACTATGAG	
*acrA*	*acrA*(+)	CCTCAAGTTAGCGGGATTAT	[26, 31]
	*acrA*(−)	ACCGTCCTGCGGGAACTTAA	

^a^(+) denotes the forward primer, and (−) denotes the reverse primer.

### 2.14. Molecular Identification of PMQR Genes

PMQR genes (*qnrA*, *qnrB*, *qnrC*, *qnrD*, and *qnrS*) were screened in all MDR‐GNB isolates using a multiplex PCR approach. Isolates yielding positive amplification signals were subsequently reanalyzed by simplex PCR to confirm gene presence. Primer sequences are provided in Table 3, and amplification conditions were applied in accordance with the methodology described by Barguigua et al. [23].

### 2.15. Molecular Identification of Disinfectant Resistance Genes

Biocide resistance genes were screened by Simplex PCR in all MDR‐GNB isolates. Targets included *qacΔE*1 and *qacE* (SMR family, quaternary ammonium resistance), as well as *CepA* and *acrA* (RND family, associated with decreased sensitivity to chlorhexidine and cationic agents). Table 3 lists the oligonucleotide primers, and PCR conditions were based on previously reported protocols [28–32].

### 2.16. Detection and Analysis of PCR Products

PCR amplicons were resolved by electrophoresis on a 1.2% agarose gel for 30 min at 100 V in 0.5× Tris‐Borate‐EDTA buffer, stained with ethidium bromide (1 *μ*g/mL), and visualized using an UV transilluminator (Cleaver Scientific Ltd, Rugby, United Kingdom). To confirm gene identity, purified PCR products were treated with ExoSAP‐IT (Thermo Fisher Scientific, Massachusetts, United States), and subjected to cycle sequencing using the BigDye Terminator v3.1 kit (Applied Biosystems, Thermo Fisher Scientific, Unites States). Sequencing was performed on a Genetic Analyzer 3130×l sequencer (Applied Biosystems, Foster City, California, United States) using the same primers listed in Table 3. Nucleotide sequences were analyzed with Chromas Pro Version 2.3 (Technelysium Pty Ltd., Tewantin, Queensland) and identified via BLAST alignment against the NCBI nucleotide database (http://www.ncbi.nlm.nih.gov/).

### 2.17. Data and Statistical Analysis

All statistical analyses were performed using IBM SPSS Statistics Version 23.0. A two‐tailed *p* value ≤ 0.05 was considered statistically significant. Prior to any inferential testing, the continuous variables were examined with the Shapiro–Wilk test to assess normality and Levene′s test to evaluate homogeneity of variances. When the data had a normal distribution and equal variances, they were analyzed with parametric tests. Otherwise, nonparametric tests were used.

Bacterial adhesion and the biofilm formation capacity across the five tested surface materials were compared with one‐way analysis of variance (ANOVA) for parametric data or the Kruskal–Wallis test for nonparametric data. When the ANOVA or Kruskal–Wallis result was significant, Tukey′s honestly significant difference (HSD) test or Dunn′s test with the Bonferroni correction was applied for pairwise comparisons.

The efficacy of disinfectants was evaluated by comparing the MIC and MBC across the isolates. Because the MIC and MBC data were not normally distributed, they were analyzed with the Kruskal–Wallis H test. When significant, Dunn′s test was employed for pairwise comparisons. Eta squared (*η* ^2^) was calculated to quantify the strength of observed differences for the MIC, MBC, and biofilm formation metrics.

The reproducibility of biofilm and disinfectant susceptibility assays was assessed based on intra‐assay and interassay coefficients of variation (CV%). To ensure reliability and consistency across the replicates, only data with a CV% of < 15% were retained for the final interpretation of the data.

The microbial loads were compared between two independent groups (e.g., direct‐contact vs. indirect‐contact surfaces) using the Mann–Whitney *U* test. This nonparametric test was used because the CFU counts showed a skewed distribution.

The categorical variables, including the ISO surface compliance status and bacterial group classifications, were analyzed using Pearson′s chi‐square (*χ* ^2^) test of independence to explore associations between contamination patterns and hospital departments, contact types, or surface categories. The exact *p* values, degrees of freedom, and chi‐square values are reported.

Principal component analysis (PCA) was performed on the MIC, MBC, and MAR index data to explore the structure of phenotypic resistance and disinfectant tolerance among the MDR isolates. It revealed interisolate similarities and underlying gradients of resistance and was used to determine which disinfectant susceptibility markers had the greatest influence on stratification of the isolates. In addition, unsupervised hierarchical clustering was conducted using Ward′s minimum variance method on pairwise distance matrices constructed from the resistance gene profiles, the ability to form biofilms, and the susceptibility to disinfectants. The resulting dendrogram shows phenotypic clusters among the MDR‐GNB isolates, which were compared with the PCA projections to examine the consistency of the clustering. The data are presented with PCA biplots, dendrograms, Sankey diagrams, and surface compliance distributions to show the associations between microbial resistance traits, surface colonization patterns, and environmental persistence in the hospital setting.

## 3. Results

### 3.1. Surface Compliance With ISO Standards

Out of the 154 environmental surfaces assessed across 13 hospital departments, 113 surfaces (73.4%) were compliant with ISO Class 6–7 microbial standards, whereas 41 surfaces (26.6%) exceeded the threshold of 10 CFU/25 cm^2^ and were considered noncompliant.

**Figure 1:**
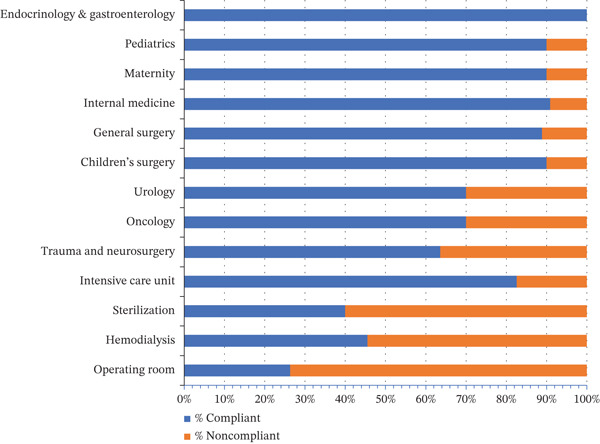
Percentage of compliant and noncompliant surfaces according to hospital department (*n* = 154). Distribution of surface compliance with ISO 6–7 standards across departments, expressed as percentage of total sampled surfaces per service.

Marked variations in compliance rates were observed between departments. The operating room recorded the highest proportion of noncompliant surfaces (73.7%), followed by the sterilization unit (60.0%) and the hemodialysis unit (54.5%). Conversely, high compliance rates (≥ 90%) were noted in departments such as endocrinology and gastroenterology, internal medicine, and pediatrics (Figure 1).

A statistically significant association was observed between hospital department and surface compliance (*χ* ^2^ = 40.49, df = 12, *p* = 0.0001), indicating that surface contamination rates differed significantly across clinical areas.

### 3.2. Microbial Load and ISO Compliance According to Surface Type and Contact Level

A total of 135 surfaces yielded quantifiable microbial counts (≤ 300 CFU/25 cm^2^) and were analyzed by surface type and level of contact. Among these, 20 surfaces were categorized as direct‐contact and 115 as indirect‐contact. The mean microbial load on direct‐contact surfaces was 7.30 CFU/25 cm^2^, compared with 5.76 CFU/25 cm^2^ for indirect‐contact surfaces. This difference was not statistically significant (Mann–Whitney *U* = 1436.0, p = 0.0754).

**Figure 2:**
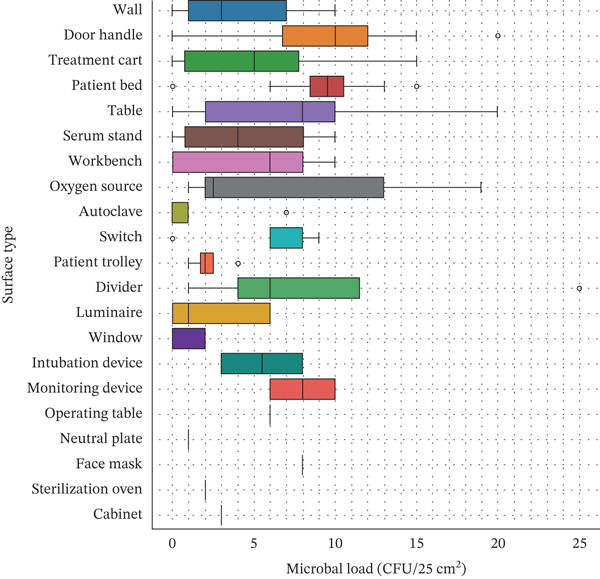
Distribution of microbial load (CFU per 25 cm^2^) across different types of hospital surfaces. Boxplots display the median (central horizontal line), interquartile range (box), and overall range (whiskers) of microbial counts measured per 25 cm^2^ of sampled surface area. Outliers are represented by individual points. Data were obtained from 154 environmental surfaces sampled in multiple hospital departments under standard aseptic conditions. Surfaces such as oxygen sources, dividers, and workbenches showed higher microbial variability, whereas sterilization ovens and cabinets remained within ISO Class 6–7 hygienic limits. This figure illustrates surface‐specific differences in microbial contamination, emphasizing the need for targeted cleaning and disinfection strategies in high‐contact areas.

When grouped by surface type, door handles exhibited the highest mean contamination (9.60 CFU/25 cm^2^), followed by dividers (9.50 CFU/25 cm^2^) and patient beds (9.17 CFU/25 cm^2^). In contrast, walls, luminaire fixtures, operating tables, and cabinet doors showed lower average loads, all below 4 CFU/25 cm^2^. Across all surface types, the overall mean microbial load was 5.99 CFU/25 cm^2^ (SD: 4.96) (Figure 2).

ISO compliance varied across surface types. Among direct‐contact surfaces, 90.0% were compliant, whereas 10.0% exceeded the ISO threshold. For indirect‐contact surfaces, 78.3% were compliant and 21.7% were noncompliant. When analyzed by surface type, patient beds (91.7% compliant) and door handles (80.0% compliant) showed high conformity despite elevated microbial loads. Other types, such as face masks (0.0% compliant) and monitoring devices (50.0% compliant), exhibited lower conformity rates.

Microbial load and ISO compliance varied across surface types and contact categories. Surfaces with direct patient interaction exhibited higher mean bacterial counts, whereas compliance rates did not consistently correspond to contamination levels.

### 3.3. Bacterial Group Distribution, Contamination Intensity, and Surface Contact Typology

Out of the 154 surface samples analyzed, bacterial growth was detected in 133 samples (86.4%), whereas 21 samples (13.6%) exhibited no detectable contamination. Gram staining and morphological classification revealed a predominance of Gram‐positive cocci (*n* = 76; 49.4%), followed by Gram‐positive bacilli (*n* = 35; 22.7%) and GNB (*n* = 28; 18.2%).

The most frequently isolated organisms were coagulase‐negative staphylococci (*n* = 52; 33.8%), *Bacillus* spp. (*n* = 35; 22.7%), *Staphylococcus aureus* (*n* = 12; 7.8%), and *Enterococcus spp.* (*n* = 6; 3.9%). Among Gram‐negative isolates, the most prevalent species included *A. baumannii* (*n* = 11; 7.1%), *E. coli* (*n* = 6; 3.9%), *E. cloacae* (*n* = 3; 1.9%), *K. pneumoniae* (*n* = 3; 1.9%), *Klebsiella oxytoca* (*n* = 1; 0.6%), *Enterobacter sakazakii* (*n* = 2; 1.3%), *Stenotrophomonas maltophilia* (*n* = 1; 0.6%), and *Providencia rettgeri* (*n* = 1; 0.6%).

Statistical analysis revealed a significant variation in the distribution of Gram groups across hospital departments (*χ* ^2^ = 121.4, *d* *f* = 24, *p* < 0.0001). GNB were predominantly isolated from critical care units, particularly ICU (*n* = 9), surgery (*n* = 8), and dialysis (*n* = 6). In contrast, Gram‐positive cocci and bacilli were more frequently distributed across internal medicine (*n* = 14), pediatrics (*n* = 15), and obstetrics‐gynecology (*n* = 12).

The spatial distribution also differed according to surface contact category. A significant association was observed between bacterial group and contact type (*χ* ^2^ = 17.3, *d* *f* = 2, *p* = 0.0002), with GNB (*n* = 28) more frequently isolated from direct‐contact surfaces, including patient beds (*n* = 9), dialysis machines (*n* = 7), and intubation devices (*n* = 5). Conversely, Gram‐positive cocci and bacilli were commonly recovered from indirect‐contact surfaces such as workbenches (*n* = 13), treatment trolleys (*n* = 10), and door handles (*n* = 9).

**Figure 3:**
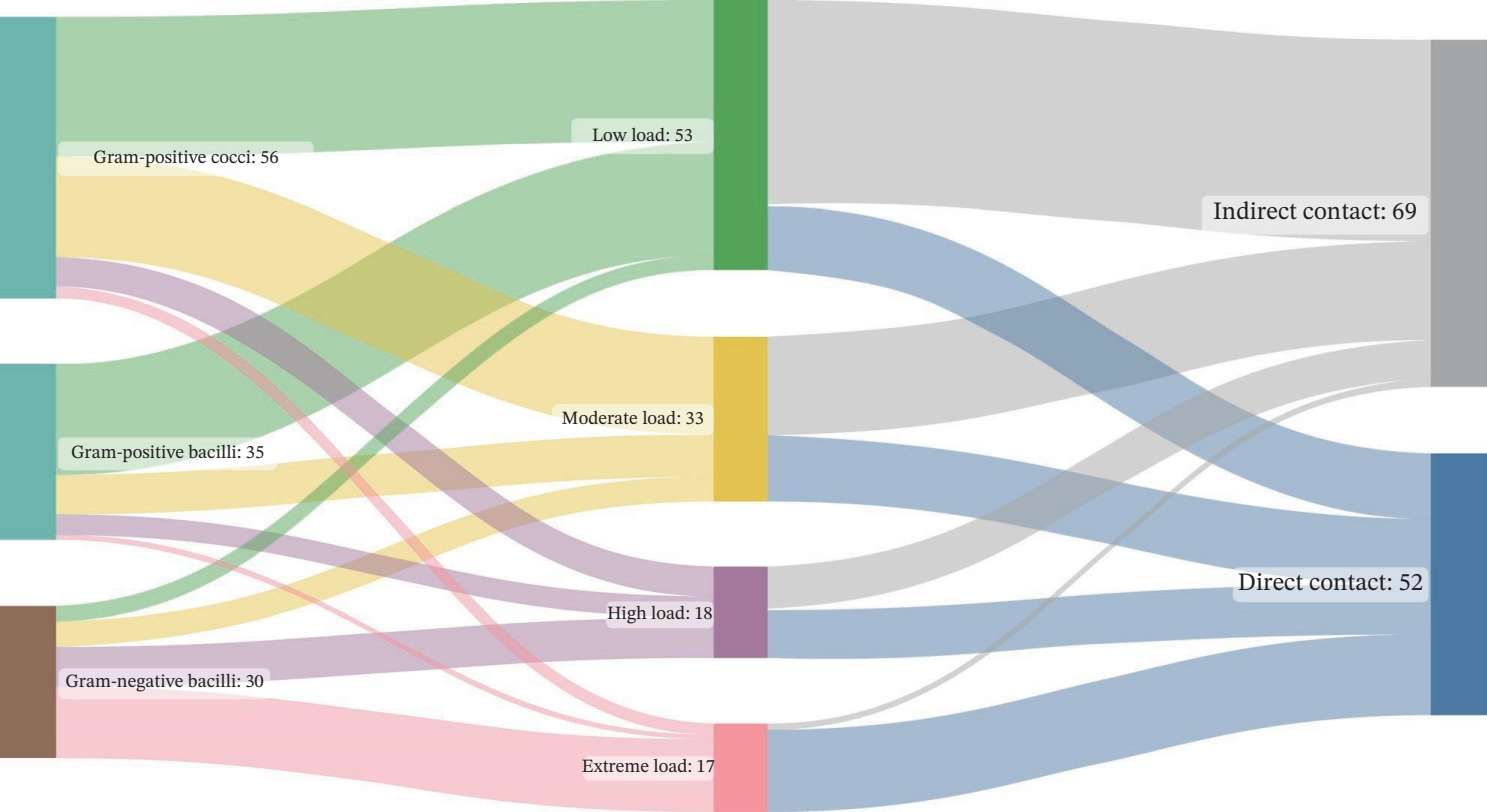
Sankey diagram depicting the distribution of bacterial groups by load intensity and contact type on hospital surfaces. Sankey diagram showing the flow of bacterial groups—Gram‐positive cocci (*n* = 56), Gram‐positive bacilli (*n* = 35), and Gram‐negative bacilli (*n* = 30)—across four contamination levels: low (≤ 5 CFU/25 cm^2^), moderate (6–25 CFU/25 cm^2^), high (26–300 CFU/25 cm^2^), and extreme (>300 CFU/25 cm^2^), and their final association with either direct‐contact (*n* = 52) or indirect‐contact (*n* = 69) surfaces. Flow width is proportional to isolate count (total *n* = 121).

To further explore contamination pathways and surface‐level microbial risk, a Sankey diagram was constructed to visualize the relationships between Gram group, microbial load class, and contact type. GNB were strongly associated with high to extreme contamination levels (CFU > 300/25 cm^2^), with 68.0% of isolates exhibiting this pattern, compared with 18.5% among Gram‐positive cocci (*χ* ^2^ = 36.27, df = 6, *p* < 0.0001). Among Gram‐positive cocci, 66.7% of isolates were linked to low or moderate microbial burdens, often associated with suboptimal but persistent contamination on low‐risk surfaces (Figure 3).

Analysis of average microbial burden confirmed this trend. Surfaces contaminated with Gram‐positive cocci exhibited the highest mean CFU counts (9.34 ± 5.41 CFU/25 cm^2^), followed by Gram‐positive bacilli (7.28 ± 5.87 CFU/25 cm^2^) and GNB (6.14 ± 4.62 CFU/25 cm^2^), with statistically significant differences between groups (Kruskal–Wallis *H* = 19.21, *p* < 0.001).

### 3.4. Antibiotic Resistance Patterns in Isolated GNB

High resistance to antibiotics was noted in the GNB study as 73.68% for piperacillin and ticarcillin, and 64.29% for tazobactam + piperacillin. It is moderate for amoxicillin + clavulanic acid (47.06%), ceftazidime (46.43%), cefepime (46.43%), amoxicillin (42.86%), sulfamethoxazole (42.86%), ciprofloxacin (39.29%), levofloxacin (39.29%), tobramycin (39.29%), gentamicin (35.71%), imipenem (32.14%), meropenem (32.14%), ceftriaxone (29.41%), aztreonam (29.41%), and amikacin (28.57%). Low resistance is noted for nalidixic acid (17.65%), ertapenem (17.65%), and cefoxitin (16.67%).

MAR index values were between 0 and 0.93, with most strains being ≥ 0.6, indicating high resistance. *A. baumannii* has MAR scores close to 0.9, suggesting widespread resistance, whereas *K. pneumoniae*, *E. cloacae*, and *S. maltophilia* had scores around 0.6 and 0.8, indicating moderate resistance. *E. coli*, *E. sakazakii*, *K. oxytoca*, and *P. rettgeri* showed weaker resistance, with MAR scores between 0 and 0.25.

**Figure 4:**
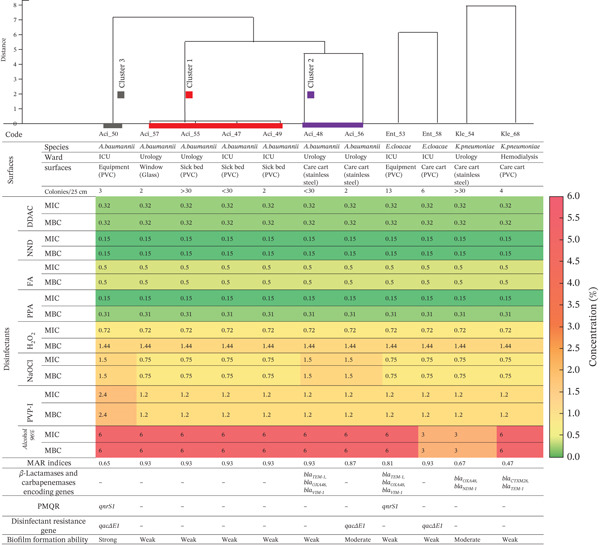
Hierarchical clustering of multidrug‐resistant Gram‐negative clinical isolates based on disinfectant susceptibility profiles and biofilm‐forming abilities. Dendrogram showing clustering of Gram‐negative isolates according to disinfectant susceptibility and biofilm capacity. The table additionally reports species, ward, surface type, colony density, MAR index, resistance genes, and biofilm formation ability. The heat map displays MIC and MBC values for eight disinfectants with color‐coded gradients. ICU, intensive care unit; PVC, polyvinyl chloride; DDAC, didecyldimethylammonium chloride; NND, N‐alkyl dimethyl benzyl ammonium chloride; FA, formaldehyde; PPA, peracetic acid; H₂O₂, hydrogen peroxide; NaOCl, sodium hypochlorite; PVP‐I, povidone‐iodine; PMQR, plasmid‐mediated quinolone resistance; MIC, minimum inhibitory concentration; MBC, minimum bactericidal concentration.

Furthermore, MDR profiles were evaluated across the isolates, revealing that 11 out of 28 strains (39.29%) exhibited resistance to two or more antibiotic classes. Among the 28 GNB isolates, the following species were identified as MDR: *A. baumannii* (63.63%; *n* = 7/11), *K. pneumoniae* (66.67%; *n* = 2/3), and *E. cloacae* (100%; *n* = 2/3). In addition, six of the 11 MDR strains were recovered from surface samples that were deemed compliant (≤ 10 CFU/25 cm^2^) (Figure 4).

### 3.5. Biofilm Formation and Genetic Determinants of MDR‐GNB Isolated

A total of 11 MDR‐GNB were recovered from hospital surfaces. Seven distinct resistance determinants were detected, including five *β*‐lactamase genes (*bla*_CTX-M-28_, *bla*_OXA-48_, *bla*_NDM-1_, *bla*_VIM-1_, and *bla*_TEM-1_), one PMQR gene (*qnrS1*), and one biocide‐tolerance gene (*qacΔE1*). Carbapenemase markers (*bla*_OXA-48_ and *bla*_NDM-1_) predominated among *β*‐lactamase genes, whereas *qacΔE1* occurred in 27.3% of isolates, mostly recovered from high‐risk units such as the ICU and urology.

Among the 11 isolates, four exhibited clinically significant *β*‐lactam resistance phenotypes—one ESBL producer and three carbapenemase producers (Figure 4). These isolates were selected for targeted molecular characterization of *β*‐lactamase genes, whereas all MDR‐GNB were screened for PMQR and biocide‐resistance determinants (*qacΔ*E1, *qac*E, *cep*A, and *acr*A).

The ESBL‐producing strain was identified as *K. pneumoniae*, isolated from a PVC care cart in the hemodialysis unit. It harbored *bla*_CTX-M-28_, exhibited a MAR index of 0.47, formed weak biofilms, and showed moderate susceptibility to disinfectants (MIC/MBC = 0.75% NaOCl, 1.2% PVP‐I, 6% alcohol).

The first carbapenemase‐producing isolate, *A. baumannii* from the stainless steel care cart in the ICU, displayed the highest MAR index (0.93) and was a weak biofilm producer. Molecular analysis confirmed the co‐occurrence of *bla*_OXA-48_ and bla_NDM-1_ along with *qnrS1* and *qacΔE1*. This isolate showed elevated MIC/MBC values for NaOCl (1.5%) and PVP‐I (2.4%), suggesting reduced disinfectant susceptibility.

The second carbapenemase‐producing isolate, *E. cloacae* from PVC equipment in the ICU ward, exhibited a MAR index of 0.81 and weak biofilm formation. It carried *bla*_TEM-1_, *bla*_OXA-48_, *bla*_VIM-1_, and *qnrS1* but lacked biocide‐resistance genes.

The third carbapenemase‐producing *K. pneumoniae*, recovered from a stainless steel care cart in the urology department, had a MAR index of 0.67, moderate biofilm formation, and carried *bla*_OXA-48_, *bla*_NDM-1_, and *qacΔ*E1.

Across all isolates, biofilm quantification revealed that eight (72.7%) were weak producers, two (18.2%) were moderate, and one (9.1%)—*A. baumannii*—exhibited strong biofilm‐forming ability. Strains with moderate to strong adherence were mainly associated with PVC or stainless steel surfaces in critical care areas, emphasizing the role of material physicochemistry in bacterial persistence.

Disinfectant susceptibility testing using MIC and MBC assays for eight commonly used agents showed heterogeneous efficacy. DDAC, NND, and FA exhibited identical low MIC/MBC values (0.32%, 0.15%, and 0.50%), indicating rapid bactericidal activity. In contrast, oxidizing and iodophoric agents (PVP‐I, NaOCl) displayed increased MICs among isolates harboring *qacΔ*E1. Kruskal–Wallis analysis confirmed statistically significant differences among disinfectants (*p* < 0.05).

### 3.6. Multivariate and Hierarchical Clustering Reveal Structured Resistance Patterns Among Hospital MDR Isolates

Multivariate analysis combining PCA, K‐means clustering, and hierarchical linkage revealed a coherent and structured phenotypic organization of 11 MDR bacterial isolates recovered from hospital surfaces. This structure was defined by a combination of disinfectant susceptibility profiles (MIC and MBC), surface colonization densities (CFU/25 cm^2^), MAR indices, resistance gene content, and biofilm formation ability.

**Figure 5:**
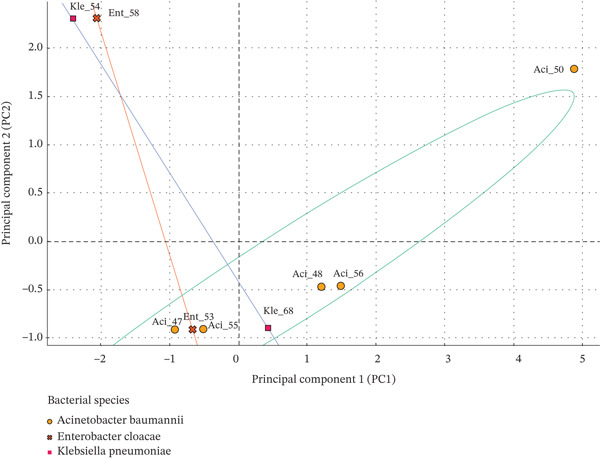
Principal component analysis reveals structuring of bacterial isolates based on disinfectant resistance and surface colonization profiles. This PCA plot summarizes phenotypic diversity among hospital bacterial isolates based on their minimum inhibitory concentrations (MIC), minimum bactericidal concentrations (MBC), colonization densities on surfaces (CFU/25 cm^2^), and multidrug resistance indices (MAR). Each point represents a unique isolate, annotated with a label combining bacterial genus abbreviation and isolate code (Aci = *Acinetobacter*, Kle = *Klebsiella*, Ent = *Enterobacter*; e.g., Aci_47 for *Acinetobacter* Isolate 47). Ellipses represent 95% confidence intervals for the distribution of each bacterial species. Reference axes (PC1 and PC2) indicate the main phenotypic gradients, particularly resistance to quaternary ammonium compounds, oxidizing agents, and alcohols.

The first two PCA components captured most of the variance in the dataset, mainly influenced by MIC/MBC values for key disinfectants (DDAC, NND, and hydrogen peroxide), MAR indices, and colonization levels. The projection did not reveal a simple resistance‐to‐susceptibility axis but rather a continuous gradient separating isolates according to their overall phenotypic profiles. *A. baumannii* and *E. cloacae* isolates such as Aci_47, Ent_53, and Aci_55 were positioned in the lower‐left quadrant of the PCA. Their clustering in this region reflects limited phenotypic divergence relative to all other isolates, as shown by their similar MIC/MBC values, consistently high MAR indices, and the absence of disinfectant‐resistance genes. In contrast, Aci_50 appeared as the most phenotypically distinct isolate, located in the upper‐right quadrant. This position corresponds to markedly higher divergence compared with the rest of the dataset, associated with strong biofilm formation, an elevated MAR index, and the presence of *qnr*S1 and *qacΔ*E1. Intermediate isolates—Aci_48, Aci_56, and Kle_68—clustered near the center of the PCA map. Their central positions reflect moderate differences compared with both the homogeneous and highly divergent groups, consistent with mixed resistance levels and intermediate phenotypic traits. Finally, Kle_54 and Ent_58 clustered in the upper‐left quadrant, showing distinct phenotypic patterns compared with the other isolates, despite weak biofilm production and the absence of biocide‐resistance genes (Figure 5).

Species‐specific variability was clearly illustrated by 95% confidence ellipses. *A. baumannii* isolates were broadly dispersed, indicating intraspecies phenotypic diversity, whereas *K. pneumoniae* isolates formed tighter, more homogeneous clusters. Isolates were annotated by species abbreviation and isolate code (e.g., Ent_53), facilitating cross‐comparison across analyses.

Unsupervised K‐means clustering (*k* = 5) confirmed the phenotypic structuring observed in PCA, identifying groups ranging from low‐divergence profiles to highly divergent environmental strains. This multivariate stratification was further refined by hierarchical clustering based on pairwise phenotypic distances. The resulting dendrogram delineated three main *A. baumannii* clusters (Figure 4):

• Cluster 1 grouped isolates Aci_47, Aci_49, Aci_55, and Aci_57, all showing identical MIC/MBC values, uniform MAR indices, absence of resistance genes, and weak biofilm production. Their pairwise distances were close to zero, confirming strong phenotypic similarity.
• Cluster 2, comprising isolates Aci_48 and Aci_56, exhibited increased antimicrobial resistance and a distinct genetic profile. Isolate Aci_48 harbored *bla*_TEM-1_, *bla*_OXA-48_, and *bla*_VIM-1_, whereas isolate Aci_56 was gene‐negative but phenotypically divergent. Both retained weak biofilm‐forming capacity and showed moderate pairwise distances (~4.7) from Cluster 1.
• Cluster 3, consisting of isolate Aci_50, was the most divergent, combining strong biofilm formation with the presence of *qnrS1* and *qacΔ*E1, and exhibited a distance > 7 from other *A. baumannii* isolates.

Among *K. pneumoniae*, two isolates were highly separated (distance = 7.92). Isolate Kle_54 harbored *bla*_OXA-48_, *bla*_NDM-1_, and *qacΔ*E1, whereas isolate Kle_68 carried *bla*_CTX-M-28_ and *bla*_TEM-1_. Both shared weak biofilm profiles but diverged in gene content and cluster assignment.

In the *E. cloacae* group, isolate Ent_53, which possessed *bla*_TEM-1_, *bla*_OXA-48_, *bla*_VIM-1_, and *qnrS1*, was distinct from isolate Ent_58, which lacked all screened genes. Although both produced weak biofilms, their molecular divergence yielded a pairwise distance of 6.08.

When comparing across clusters and species, isolates in low‐divergence clusters (e.g., Aci_47, Aci_55, and Ent_58) showed minimal antimicrobial and biocide resistance, absence of resistance genes, and weak biofilm formation. In contrast, isolates in highly divergent clusters (e.g., Aci_50, Ent_53, and Kle_68) accumulated multiple resistance determinants (e.g*., bla*_VIM-1_ and *qacΔ*E1) and frequently exhibited enhanced biofilm formation (Figure 4).

### 3.7. Material‐Specific Biofilm Formation

**Figure 6:**
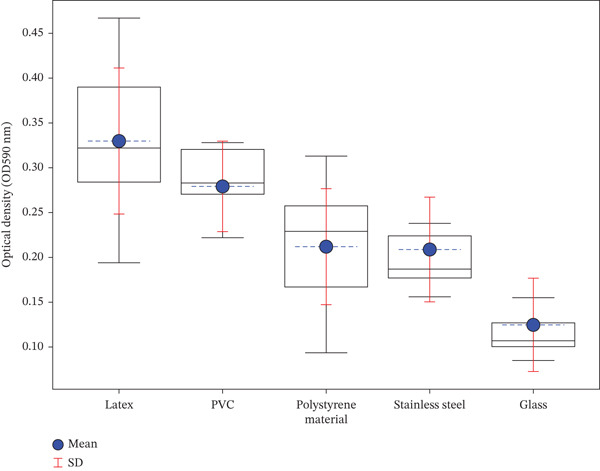
Optical density of biofilms formed by MDR‐GNB on various materials; PVC, polyvinyl chloride. Boxplots represent the distribution of OD values for each material: latex, PVC, polystyrene, stainless steel, and glass. The blue circles indicate the mean OD for each material, with dashed lines showing the mean value, and red error bars represent the standard deviation (SD).

**Figure 7:**
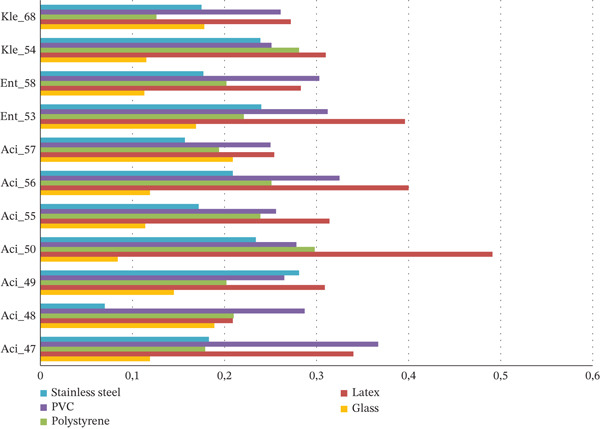
Optical density‐based comparison of MDR strain adhesion on various surface materials; PVC, polyvinyl chloride. This bar plot illustrates the adhesion level (OD₅₉₀) of each bacterial isolate on polystyrene, PVC, latex, glass, and stainless steel. Strain labels combine genus abbreviation and isolate code (Aci = *Acinetobacter*, Kle = *Klebsiella*, Ent = *Enterobacter*; e.g., Aci_47 *for Acinetobacter* Isolate 47). PVC, polyvinyl chloride.∗.

The evaluation of bacterial adhesion across different materials revealed distinct material‐dependent profiles. Mean OD values (OD590nm ± SD) showed significant variations among materials. Latex surfaces exhibited the highest mean OD values (0.330 ± 0.077), followed by PVC (0.285 ± 0.032). Intermediate adhesion levels were observed on polystyrene (0.210 ± 0.053) and stainless steel (0.209 ± 0.058), whereas glass surfaces showed the lowest adhesion capacity (0.129 ± 0.052) (Figure 6). Statistical analysis confirmed significant differences between materials (one‐way ANOVA, *p* < 0.001). Levene′s test validated the homogeneity of variances between groups (*p* > 0.05), and Tukey′s post hoc analysis revealed significant differences (*p* < 0.05) between glass and both latex and PVC surfaces. This material‐dependent hierarchy in bacterial adhesion (latex > PVC > polystyrene ≥ stainless steel > glass) demonstrates the substantial influence of surface properties on bacterial adhesion capacity. In addition, strain–material interaction was significant, with the strong biofilm producer *A. baumannii* strain Aci_50 showing enhanced adhesion on latex and PVC compared with other materials. Even weak producers demonstrated increased biofilm formation on latex and PVC surfaces. All strains consistently showed minimal adhesion to glass, suggesting its superior resistance to bacterial colonization (Figure 7). Statistical analysis confirmed significant differences between materials (*p* < 0.001), particularly between glass and higher adhesion materials (latex, PVC).

## 4. Discussion

This study combined microbiological, molecular, and statistical approaches to characterize MDR‐GNB colonizing hospital surfaces in a Moroccan healthcare facility. In total, 154 environmental samples were collected from 13 hospital departments and subjected to quantitative microbiological analysis, phenotypic screening for antibiotic and disinfectant resistance, and molecular detection of key resistance genes (*bla_NDM_*, *bla_OXA-48_*, *qnrS*, *qacΔ*E1, and *acrA*). Moreover, the ability of these isolates to adhere to and form biofilm on glass, PVC, latex, and stainless steel was examined.

The data revealed widespread colonization of hospital surfaces by MDR‐GNB, highlighting a disconnect between traditional microbial surveillance standards and the actual clinical risk. Although the majority of the surfaces (73.4%) adhered to the ISO Class 6–7 microbial thresholds, several could harbor MDR‐GNB isolates carrying clinically significant resistance genes, including *bla*_NDM_, *qnrS1*, and *qacΔ*E1. This finding questions the validity of using absolute CFU counts as the sole marker of compliance to hygiene regulation. In this line, Shams et al. reported MDR organisms on hospital surfaces at very low levels, ranging from ≤ 1 to 524 CFU/100 cm^2^ in terminally cleaned rooms [33]. Indeed, the isolation of MDR strains from surfaces with low contamination emphasizes that microbial virulence and resistance potential are not necessarily correlated with colony abundance.

Recent studies have shown that MDR‐GNB, including *K. pneumoniae*, can persist on dry hospital surfaces for several months (up to 600 days in some instances); thus, there is a long‐term risk of transmission in clinical settings [34]. This resilience is partly due to their ability to form dry‐surface biofilms, which protect against desiccation and reduce susceptibility to cleaning agents [35]. This finding aligns with recent calls for a paradigm shift toward risk‐based surface evaluation that incorporates molecular markers of resistance and virulence [36–38]. Environmental screening should not focus exclusively on bioburden; it should also consider the presence of high‐risk clones, particularly those that carry plasmid‐borne resistance determinants. The use of targeted qPCR or metagenomic tools may significantly enhance the early detection of such threats in clinical settings [39, 40].

There were marked differences in the contamination levels across the hospital units. Specifically, the operating room and sterilization zone, where invasive devices are frequently manipulated, presented the highest rates of surface noncompliance and MDR detection. A combination of relatively high patient turnover, complex procedures, and suboptimal biocide rotation strategies can explain these findings [41–43]. Interestingly, MDR isolates were also found in the endocrinology and gastroenterology units, which are not typically associated with high microbial risk. This finding might be explained by latent reservoirs and indirect transmission routes, possibly involving healthcare personnel and/or shared equipment [7, 44]. Hence, there is a need for strong localized infection risk models that consider patient vulnerability, antimicrobial exposure, and surface functionality rather than the specific department [45].

Surface type also emerged as a key determinant of microbial burden. Surfaces to which patients and healthcare personnel are in constant contact, such as door handles, infusion pumps, and bed rails, exhibited higher CFU counts and a greater diversity of resistant isolates. MDR‐GNB were also detected on surgical masks and medical trolleys, which can often be overlooked as sources of contamination. These findings underscore the importance of not just focusing on the disinfection of static surfaces.

The physicochemical properties of a surface, notably its hydrophobicity and porosity, influence the ability of bacteria to adhere to it and form biofilm. In this study, there was greater biofilm formation on the hydrophobic materials (latex and PVC) compared with the hydrophilic surfaces (glass). Consistently, researchers have shown that surface‐free energy and roughness contribute to the initial attachment of microbes and their long‐term persistence [22, 46, 47]. The finding that isolates with high MAR and biofilm indices were predominantly associated with PVC and stainless steel surfaces is a novel ecological association that links surface physicochemistry to the maintenance of multidrug resistance. Such material‐specific risk factors should be considered in hospital procurement policies, particularly for critical care settings. There are several options to mitigate long‐term contamination, including the incorporation of antimicrobial‐coated materials or the replacement of high‐risk surfaces with more inert alternatives [48]. It is important to remember that there is no one‐size‐fits‐all solution: Decisions must be based on robust environmental surveillance data and the specific characteristics of each institution.

This study demonstrated that the interplay between biofilm formation and antimicrobial resistance is a defining trait of environmental MDR‐GNB. Most of the isolates were weak biofilm producers, but those that formed strong biofilms exhibited higher MAR indices and carried a broader spectrum of resistance genes, including *bla*_OXA-48_, *bla*_NDM_, *qacΔE1*, and *acrA*. These findings add to a growing body of evidence that biofilms serve as a protective niche enabling horizontal gene transfer, antibiotic sequestration, and metabolic quiescence, all of which act synergistically to enhance multidrug tolerance [49, 50]. Of note, the biofilm phenotype was not the same for all tested surfaces. Biofilm biomass was significantly higher on PVC and latex, which are commonly found in medical tubing, gloves, and bedrails, than on glass. Hence, the choice of material affects the initial ability of microbes to adhere to it and also determines the resilience of the biofilms that are established [51, 52]. This has direct implications for infection control: recurrent environmental contamination and the persistence of MDR clones may be enhanced on surfaces that promote biofilm formation, especially in humid or poorly ventilated hospital microenvironments.

At the molecular level, there were several novel genotype–phenotype associations. Carbapenemase genes (*bla_OXA-48_* and *bla_NDM-1_*) co‐occurred with the biocide‐resistance determinant *qacΔE1* in isolates from the ICU and urology units, suggesting genetic and phenotypic convergence between antibiotic and disinfectant tolerance. Interestingly, some isolates harbored *qnrS1* and carbapenemase genes (*bla_NDM-1_* and *bla_VIM-1_*), indicating the environmental dissemination of PMQR. This phenomenon has rarely been documented on hospital surfaces within Moroccan healthcare settings. Consistently, there is accumulating evidence that exposure to subinhibitory concentrations of disinfectants—especially chlorhexidine and quaternary ammonium compounds—can select for microbial populations that tolerate biocides and present multidrug resistance, often via broad‐spectrum efflux mechanisms [53]. Based on experimental adaptation studies, such strains may also exhibit reduced susceptibility to antibiotics like fluoroquinolones, daptomycin, and *β*‐lactams [54, 55]. These findings emphasize the importance of biocide stewardship in preventing indirect selection of antibiotic resistance.

Beyond structural resistance, the convergence of biocide tolerance and antibiotic resistance reflects adaptive synergy that could be reinforced by selective pressure from the overuse of disinfectants [56, 57]. Certain MDR‐GNB strains may persist in hospital environments due to the ability to combine adaptive colonization of specific materials, cross‐resistance to biocides and antibiotics, and biofilm‐mediated protection. Collectively, these patterns delineate a novel ecological framework for understanding environmental MDR persistence. This triad represents a formidable challenge to current cleaning protocols and highlights the importance of integrated decontamination approaches that address all three layers of resistance.

Multivariate analyses also provided evidence of adaptive convergence, with clustering of unrelated species that share similar resistance, biofilm, and surface‐association profiles. This cross‐species alignment of survival strategies under comparable selective pressures has rarely been reported for surface microbiology [58]. Multivariate clustering methods have proven to be particularly powerful in environmental microbiology to trace transmission between patients, surfaces, and healthcare workers. For instance, a recent study conducted in an ICU applied principal coordinate analysis and hierarchical clustering analyses, revealing the ICU environment as a major reservoir of antimicrobial resistance, with patients′ oropharyngeal swabs carrying the highest antimicrobial resistance burden. Although healthcare staff contributed to the spread of MDR organisms, they were not primary sources, and the MDR paterns in their microbiomes closely resembled those observed in environmental samples [59]. Multivariate models have also been applied to environmental metagenomic datasets to link microbial community structures with surface properties and cleaning regimes [60]. The findings from data‐driven clustering offer important insights for infection control. Isolates from high‐risk clusters—high MAR indices, strong biofilm formation, and tolerance to disinfectants—were recovered much more frequently from critical care units (e.g., ICUs and hemodialysis services). These multivariate signatures can be used to predict which reservoirs should be monitored more closely. The data presented here could be used to develop novel panels with a small number of markers to rapidly screen these areas. For example, the codetection of *qacΔ*E1, *bla*_NDM_, and a high OD biofilm phenotype could serve as a proxy to identify persistent MDR hotspots. It is necessary to validate such panels against clinical outcomes before they can be implemented into routine hospital hygiene assessments.

Taken together, this study has established an integrated ecological model of how resistance is maintained in healthcare environments. The findings have revealed new links between genetic determinants (*bla_OXA-48_*, *bla_NDM-1_*, *qnrS1*, and *qacΔ*E1), the physicochemical properties of surface materials, and phenotypic persistence traits.

## 5. Study Limitations and Future Perspectives

### 5.1. Study Limitations

While this investigation presents the first integrated microbiological, molecular, and statistical analysis of MDR‐GNB colonizing hospital surfaces in a Moroccan facility, it has some constraints. The single‐center, cross‐sectional design limits generalisability and temporal resolution; absence of whole‐genome sequencing precludes detailed insight into plasmid‐mediated transmission; biofilm assays under laboratory conditions may not fully replicate hospital surface dynamics.

### 5.2. Future Perspectives

We recommend longitudinal, multicentre environmental studies including whole‐genome analyses and plasmid tracking; development of in situ biofilm models on actual hospital materials subject to routine cleaning cycles; and intervention trials comparing standard and risk‐based surface‐hygiene protocols incorporating molecular screening and material‐design optimisation. These avenues will further our understanding of MDR‐GNB persistence and support evidence‐based improvements in hospital infection‐prevention strategies.

## 6. Conclusion

This study combined phenotypic, molecular, and multivariate approaches to provide the first integrated analysis of MDR‐GNB colonizing hospital surfaces in a Moroccan healthcare facility. The results revealed that 26.6% of the examined surfaces exceeded the ISO Class 6–7 microbial thresholds. In addition, several low‐burden sites still harbored MDR isolates carrying critical resistance genes such as *bla*_NDM_, *bla*_OXA-48_, *qnrS*, and *qacΔ*E1. The persistence of these strains was strongly associated with biofilm formation and the type of material. Specifically, hydrophobic surfaces such as latex and PVC favored adhesion and biomass accumulation. Isolates carrying both biocide‐ (*qacΔ*E1 and *acr*A) and antibiotic‐resistance genes demonstrated higher MIC and MBC values for both disinfectants and antimicrobials. These findings underscore the coselection pressures acting in hospital environments. Multivariate analyses (PCA and hierarchical clustering) revealed distinct phenotypic signatures linking biofilm production, surface adhesion, and resistance gene content, providing a novel framework for environmental risk profiling in healthcare settings.

Overall, these findings emphasize that surface contamination cannot be evaluated solely by colony counts; it must also incorporate molecular and ecological indicators of risk. This approach could involve integrated surface surveillance systems that combine microbiological monitoring, rapid gene detection, and material‐specific risk assessment to improve infection prevention strategies. Future studies should validate these molecular–ecological signatures in other hospitals and evaluate interventions targeting high‐risk materials and biofilm‐prone surfaces.

## Funding

No funding was received for this manuscript.

## Ethics Statement

The study was conducted in accordance with the Declaration of Helsinki and approved by the Comité d′Éthique pour la Recherche Biomédicale d′Oujda (CERBO), affiliated with the Faculty of Medicine and Pharmacy, Mohammed First University, Oujda, Morocco (Approval No.: 22/2021).

## Conflicts of Interest

The authors declare no conflicts of interest.

## Data Availability

The data that support the findings of this study are available from the corresponding author upon reasonable request.
